# Plutonium in the Arctic Marine Environment — A Short Review

**DOI:** 10.1100/tsw.2004.100

**Published:** 2004-06-18

**Authors:** Lindis Skipperud

**Affiliations:** Department of Plant and Environmental Science, Agricultural University of Norway, Norway

**Keywords:** plutonium, sources, species, isotope ratios, arctic marine environment

## Abstract

Anthropogenic plutonium has been introduced into the environment over the past 50 years as the result of the detonation of nuclear weapons and operational releases from the nuclear industry. In the Arctic environment, the main source of plutonium is from atmospheric weapons testing, which has resulted in a relatively uniform, underlying global distribution of plutonium. Previous studies of plutonium in the Kara Sea have shown that, at certain sites, other releases have given rise to enhanced local concentrations. Since different plutonium sources are characterised by distinctive plutonium-isotope ratios, evidence of a localised influence can be supported by clear perturbations in the plutonium-isotope ratio fingerprints as compared to the known ratio in global fallout. In Kara Sea sites, such perturbations have been observed as a result of underwater weapons tests at Chernaya Bay, dumped radioactive waste in Novaya Zemlya, and terrestrial runoff from the Ob and Yenisey Rivers. Measurement of the plutonium-isotope ratios offers both a means of identifying the origin of radionuclide contamination and the influence of the various nuclear installations on inputs to the Arctic, as well as a potential method for following the movement of water and sediment loads in the rivers.

### SOURCES OF CONTAMINATION

Radioactivity has both natural and anthropogenic sources. The natural radiation stems from the decay of nuclei in the Earth’s crust and from cosmic radiation. In addition to this natural radiation, human activities have added radionuclides like plutonium (Pu) to the Arctic environment. This paper will give a short overview when it comes to Pu in the Arctic environment.

### Weapon-Related Sources

#### Weapon Production

Russia has three fuel reprocessing plants that started out as weapon production facilities at Mayak, Krasnoyarsk, and Tomsk, which are situated south of the Arctic. But radioactive releases from the Mayak and Tomsk installations enter the drainage area of the Ob River and those from Krasnoyarsk go directly into the Yenisey River. They can eventually be transported to the Kara Sea. This paper will go into the problems surrounding Mayak and the Kara and Irish Sea.

#### Nuclear Weapon Tests

Past atmospheric tests of nuclear weapons remain by far the largest global source of radioactive contamination. In all, 520 atmospheric nuclear test detonations have taken place, the major part by the U.S. and the former USSR[1]. Most of the atmospheric tests were done before 1963, about 25% in the years of 1957 and 1958 and 75% in 1962 and 1963, and atmospheric testing ceased entirely in 1980. Novaya Zemlya, Russia, was the only site for atmospheric nuclear weapon tests in the Arctic. No such tests have been conducted there since 1962. About 0.33 PBq $^{238}$Pu, 6.5 PBq $^{239}$Pu, 4.3 PBq $^{240}$Pu, and 142 PBq $^{241}$Pu were released by past atmospheric nuclear tests[1,2].

Some underground nuclear tests also took place in the Arctic at Novaya Zemlya, Russia, and at Amchitka Island, Alaska.

Global fallout of nuclear weapon debris was only deposited directly into the Arctic to a small degree; the main fraction of fallout radioactivity has been transported from the North Atlantic[3].

#### Underwater Weapon Tests

Chernaya Bay is a fjord inlet, connected to the Barents Sea, on the southwestern coast of Novaya Zemlya. The former Soviet Union used the bay to conduct underwater tests of nuclear bombs in 1955 and 1957, and in the vicinity of the bay in 1961. As a result, the bottom sediments there are contaminated with elevated levels of Pu, as well as other radioactive isotopes.

Today, the inventory of Pu in Chernaya Bay is similar to other sites of major Pu contamination, such as the most contaminated areas in the Irish Sea in the vicinity of the Sellafield reprocessing plant.

#### Sunken Nuclear Submarines

In 1989, the Russian nuclear submarine *Komsomolets* caught fire and sank southwest of Bear Island in the Norwegian Sea and, for an unknown reason, the nuclear submarine *Kursk* sank in the year 2000. The submarines contained nuclear reactors and torpedoes with mixed uranium-Pu warheads. Small amounts of radionuclides have leaked out of the *Komsomolets*, but the likelihood of a large-scale release from the submarines is small. The uranium and Pu will be released with time as the casing is breached. However, Pu has high affinity to particles and will most likely be retained in the sediments close to the wrecks.

#### American Aircraft Crash

In January 1968, an American B-52 aircraft carrying four nuclear weapons crashed on the ice in Bylot Sound near Thule, Greenland. The impact trigged conventional explosives, which led to fragmentation of the nuclear weapons on board and spread the Pu over the ice. Even after cleanup, approximately half a kilogram (≈1.4 TBq) of Pu sank to the underlying sediments[4].

### Civilian Use of Nuclear Explosives

#### *Production of Electricity, Operational Releases*

There are a lot of nuclear power plants (NPPs) around the world, which can give rise to contamination of the environment directly, through routine releases from nuclear reactors onto cooling water streams. For the Arctic area, the NPPs at Kola (near Plyarny Zori on the Kola Peninsula) and the Bilibino NPP, which is in the Chukchi region of eastern Russia, and NPPs in Sweden, Finland, and Russia are of special concern. Also of concern for the Arctic region are the NPPs in Sweden, Finland, and Russia that are within 1000 km of the Arctic Circle.

#### *Reprocessing Plants*

Spent nuclear fuel is often processed to recover U and Pu. Water released to the sea in reprocessing contains a soup of different radionuclides and some of this contaminated water has been released routinely into the sea. In Europe, three reprocessing plants are relevant to the Arctic because of transport of radionuclides by ocean currents: Sellafield in Cumbria on the northwest coast of England, La Hague near Cherbourg, France, and Dounreay in northeast Scotland.

Sellafield has been the most important source of radionuclides to the Arctic marine environment because of the scale of its discharge. The effluent has been released into the sea and carried north by ocean currents. The yearly average releases from Sellafield during 1973–1976 were 0.011 PBq $^{238}$Pu, 0.04 PBq $^{239,240}$Pu, and 1.16 PBq $^{241}$Pu[5]. Total releases for the period 1951–1992 amounts to 0.12 PBq $^{238}$Pu, 0.61 PBq $^{239,240}$Pu, and 21 PBq $^{241}$Pu[6]. Releases from La Hague are not as well documented, but releases of Pu from La Hague amounts to about 0.4% of the Sellafield releases[7].

#### *The Chernobyl Accident*

On April 26, 1986, two explosions in quick succession blew the roof off one of the four reactors in the Chernobyl NPP in the Ukraine, leaving a gaping hole that exposed the reactor core. During a ten-day fire, smoke and gases rose more than 1 km into the atmosphere, followed by fragments of uranium fuel. Alpha activity from the Chernobyl accident was dominated by the $^{242}$Cm, but this is of no importance because of the short half-life ($T_{½}$ = 163 days). It is estimated that about $0.1 \times 10^{15}$ Bq of the release was alpha-emitting Pu nuclides, but most of these were deposited in the vicinity of Chernobyl and will therefore not be discussed in this paper. The fallout of Pu from Chernobyl into the Baltic Sea is estimated to be 1.5 and 0.72 TBq of $^{239,240}$Pu and $^{238}$Pu, respectively, approximately 99% of which is assumed rapidly transferred to the sediment. Deposition in the Baltic Sea of $^{239,240}$Pu and $^{238}$Pu amounts to about 10 and 100% of the global fallout content, respectively[3].

#### *Mining and Construction Work*

The former Soviet Union used nuclear explosives to carry out mining and construction work from 1967–1988 [1]. At three of the sites, accidents led to significant local contamination.

#### Dumping of Nuclear Waste

The Soviet Union dumped high-, intermediate-, and low-level radioactive waste in the Arctic Seas (solid waste in Kara Sea and in fjords of Novaya Zemlya, liquids in the open Kara and Barent Seas) during the years 1959–1991, including six nuclear submarine reactors and a shielding assembly from an icebreaker reactor containing spent fuel. At the time of dumping, experts estimated that the spent nuclear fuels represented a total activity of $8.5 \times 10^{16}$ Bq[8,9,10,11].

### PU BEHAVIOUR IN THE ENVIRONMENT

Of the artificial radionuclides released to the environment by nuclear activities, the transuranium elements, such as Pu, are a major concern. This concern arises from the long half-lives, their accumulation in bone, as well as the high radiotoxicity. Pu has been produced in greater quantity than other transuranic elements, however, environmental assessments are complicated by the complex environmental behaviour of the element. This derives from the existence of at least four oxidation states in aqueous environments[12] and that Pu in the different oxidation states can form relatively stable complexes under various environmental and biological conditions[13].

For multivalent elements like Pu, releases from dumped nuclear waste may contain Pu included in corroded particles, Pu-colloids, oxidised forms ($Pu^{V,VI}$), reduced forms ($Pu^{III,IV}$), or organic complexes due to interaction of Pu-species with degraded products of the protective barrier. The behaviour of Pu in the sediment-seawater system depends on the speciation as corrosion particles and aggregating colloids may settle, while the retention of mobile Pu-species in sediments may be negligible (Fig. 1).

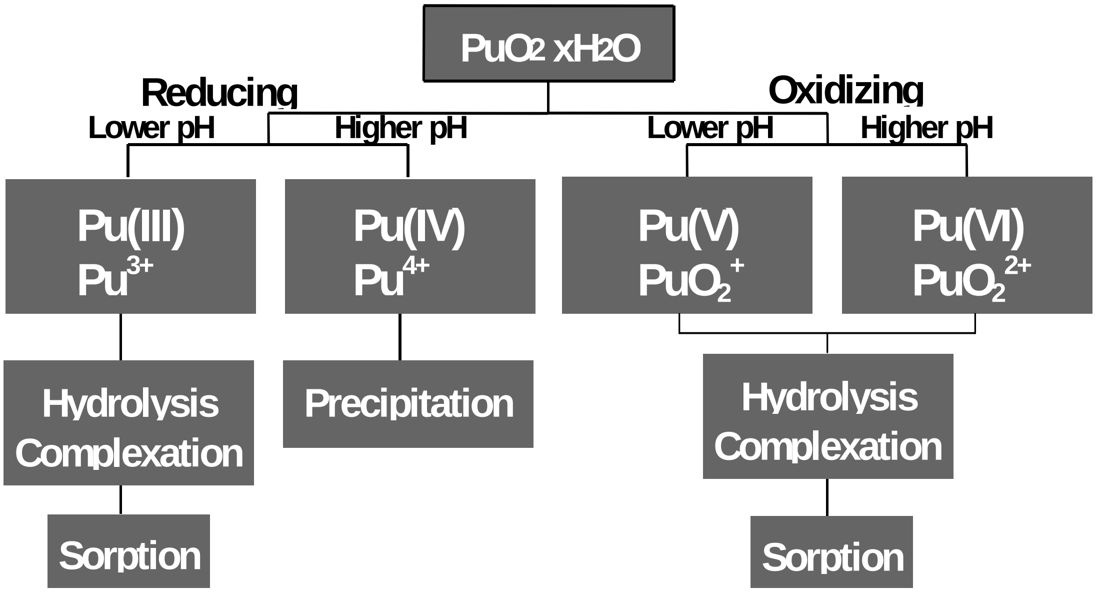

**FIGURE 1.** Speciation diagram for the range of reactions to be considered in studying the environmental behaviour of Pu[14,15].

In Sellafield, releases have shown differences in chemical speciation such as particles and colloids[16], as well as different oxidation states. Reduced Pu(III,IV) is present and predominating in the sea tank effluent and oxidised Pu(V,VI) is predominating in the filtrate of the effluent from the cooling ponds. In view of the small contribution of the pond effluents to the total Pu discharges, only about 1% of the total would appear to be in an oxidised form[17].

For well-oxygenated waters, most of the true "dissolved phase" Pu has been identified as Pu(V)[13,18,19,20]. This is supported by new data from the northwest coast of Greenland, near the Thule accident site[21]. This shows that Pu(V,VI) must be quite stable when reaching the water, as in the Irish Sea, and can be transported long distances by the water masses. $PuO_2^+$ has a low tendency to hydrolysis and complexation and is less likely to be sorbed to solid surfaces and to colloidal particles than the other oxidation species. As a consequence, Pu can be expected to migrate most rapidly if it is in the pentavalent oxidation state (Table 1).

**TABLE 1**
**Oxidation States of Pu in Marine and Natural Water[14,15,22]**

| Ox. State | Ion | Stability |
|---|---|---|
| Pu(III) | $Pu^{3+}$ | Unlikely to exist in oxic waters, but may be present in anoxic waters. Oxidation to $Pu^{4+}$. |
| Pu(IV) | $Pu^{4+}$ | Hydrolysis. Hydrolysed Pu(IV) sorbs on colloidal and suspended material. Formation of $Pu(OH)_{4\ (am)}$ in marine and natural waters. |
| Pu(V) | $PuO_2^+$ | Most stable oxidation state in marine and natural oxic waters. |
| Pu(VI) | $PuO_2^+$ | Easily reduced to $PuO_2^+$. |

In marine and natural waters, the limiting solubility is associated with either the carbonate or the hydroxide, depending on the oxidation state, pH, and carbonate concentration. In this respect, the presence of carbonate is known to increase the solubility of Pu. Pu solubility is also limited by the formation of $Pu(OH)_4$(amorphous). Moreover, hydrolysed Pu(IV) sorbs on colloidal and suspended material, both inorganic and biological, therefore redox and hydrolysis reactions result in the sorption of Pu to sediments.

The total solubility is limited by the formation of the highly insoluble $Pu(OH)_4$. The sorption of hydrolysed Pu(IV) in neutral water on mineral and organic-coated surfaces is accountable for the very low concentrations of dissolved Pu, even in the absence of $Pu(OH)_4$ and $PuO_2^+$. Desorption is accomplished only by strong complexing and/or redox reagents. The intractable nature and strong tendency of $Pu(OH)_4$ to sorb on surfaces is a dominant and often controlling feature of Pu geochemistry[23].

Since the 1970s, scientists have stressed the importance of Pu-oxidation states in determining the environmental chemistry[18,24,25] and biological availability of Pu[26]. The way in which Pu partitions itself between possible species in any given environmental system is known to depend on the chemical form in which it enters the system, redox conditions, pH, ionic strengths, the type and concentration of organic and inorganic complexing agents, and the extent of hydrolytic colloid formation[23,27].

The order of distribution coefficient is that of Am and Cm > Pu > Np, although the Pu value will certainly be that for mixture of oxidation states. It has been shown that the distribution coefficients for Pu(III,IV) ($10^6$) exceed those for Pu(V,VI) (less than $10^4$) by more than two orders of magnitude in a marine system[18]. Furthermore, the interaction of Pu-species with soils and sediments is a time- and temperature-dependant process. Thus, the distribution coefficient, Bq/kg sediment per Bq/l sea- or freshwater, increases with time until pseudoequilibrium/equilibrium is reached ($K_D$). Thus, the contact time between contaminated seawater and sediments, e.g., the rate of a contaminated "plume", should be taken into account in dispersion and dose assessment models[28].

### PU IN MAYAK

The “Mayak” Production Association (Mayak PA) was established in the late 1940s to produce Pu for the Soviet nuclear weapons program[29]. The site is located at the head of the Techa River in the Southern Urals and comprises military and civil reactors as well as reprocessing and metallurgical plants[30].

At Mayak PA, three major incidents have released radioactive nuclides in to the environment:

- Discharges of liquid radioactive wastes to the Techa River (1949–1956)
- The Kysthym accident; releases due to an explosion in a high-level radioactive waste tank (1957)
- Wind transport of contaminated sediments from Lake Karachay (1967)

The Techa River is part of the hydrographic system of the Ob River and is a tributary of the Iset River. The river system Techa – Iset – Tobol – Irtysh – Ob discharges into the Kara Sea, hence is an important transport route of radioactivity from the Urals to the Northern Seas. The Techa River walley contains about 40-km length of boggy, slightly sloping flood plain between 200–1000 m wide. This is the so-called Asanov Swamp without any clearly defined river channel, the river winds, and the current is slow (Fig. 2)[29].

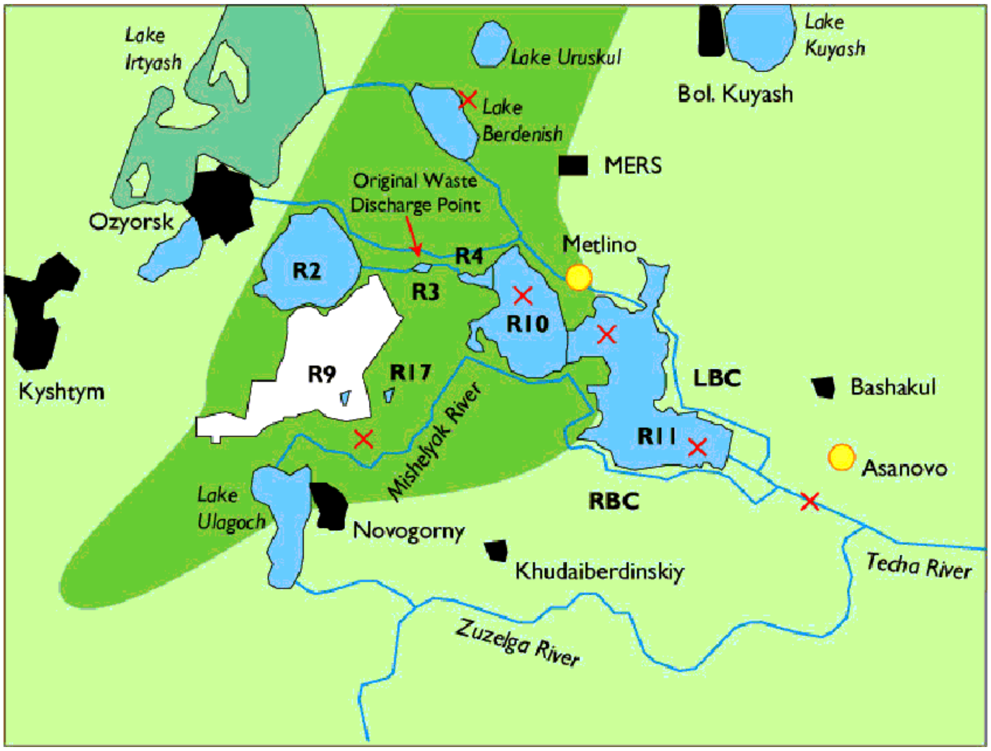

**FIGURE 2**. A map showing reservoirs and lakes in the Mayak area (x showing some of the sampling sites).

#### Inventory

The Pu concentrations in the Mayak area vary due to the different accidental releases and the distance from the outlet. History releases and some concentrations are given in Table 2. For soil, the activities for total Pu are given in Table 3.

**TABLE 2**
**Brief History[29] Together with Major Routine and Accidental Releases of Beta-Emitting Radionuclides from Mayak PA (PBq = $10^{15}$ Bq)[30]**

| | |
|---|---|
| June 1948 | First military uranium-graphite reactor operational; reprocessing and chemical/metallurgical plants completed. |
| **1948–1956** | **Discharge of 106 PBq Intermediate Level Waste to the Techa River (98% from Dec 1949 to Nov 1951). Total α discharges amounted to about 0.44 TBq/year in 1949, 0.81 TBq/year in 1950, and 0.74 TBq/year in 1951.** |
| August 1949 | High-purity metallic Pu components produced for the first Soviet atomic bomb; exploded 29th August 1949 at Semipalatinsk, Kazakhstan. |
| 1949–1955 | Five more military reactors constructed. |
| **1951–** | **Discharge of 20,000 PBq Intermediate Level Waste to Lake Karachay. Concentrations of total α emitters in water of Lake Karachay from 1970–1993 (spring sample collection) is as follows: 0.13 MBq/l in 1970, 0.010 MBq/l in 1975, 0.037 MBq/l in 1980, 0.32 MBq/l in 1985, 0.22 MBq/l in 1990, 0.63 MBq/l in 1993.** |
| 1956 | Dam 10/Reservoir 10 complete. |
| **1957** | **Accidental release and dispersal of 740 PBq radionuclides after an explosion in a high-level waste tank - Kysthym Accident. 90% settled in the immediate vicinity of the explosion site, the remaining 74 PBq was dispersed by the wind and caused the radioactive trace (EURT) along the path of the plume. Transuranic had been removed prior to storage.** |
| 1963 | Dam 11/Reservoir 11 complete. |
| **1967** | **Wind dispersal of 0.044 PBq–contaminated sediments from Lake Karachay. Most of this contained Cs-137, Sr-90 and Y-90, Ce-144 and Pr-144, and almost nothing was α-emitting nuclides.** |
| 1977 | New reprocessing plant for civil fuel completed. |
| 1979 | Seventh reactor in operation. |
| 1987 | First military reactor shut down after 39 years of operation. |
| | Production of weapons grade Pu ceased. |
| 1987–1991 | Four other reactors shut down. |
| 1992– | Two remaining reactors producing radionuclides for civil and military use. |

Pu concentrations in groundwater collected from boreholes varied from 0.03–2.8 Bq/l. There were variations both between the wells and in sampling depth. Pu activity levels in water from Reservoirs 10 and 11 were found to be 0.002–0.08 Bq/l, while the activity levels in sediments vary due to distance from source and core depth, with the highest average activity levels in Reservoir 10. Mean sediment contamination densities are given in Table 4. Fig. 3 shows only the $^{239}$Pu activity in surface sediments and filtered water downstream Techa, Iset, and Ob Rivers.

The flux of radioactive materials in the Techa River is now steadily decreasing. The influence on the radioactive contamination of the Kara Sea is unknown, but it is considered insignificant under the present situation. The main concern in the future is the remobilisation of radionuclides from soils and river sediments. As far as we know, the soils and sediments might act as a diffuse source in the future, giving rise to increased radioactivity concentration in the Kara Sea.

### Isotopic Ratios

Pu does not represent the major health risk in the area at present; activities of $^{137}$Cs in Techa River soils and sediments and $^{90}$Sr and $^{60}$Co in surface and groundwaters are between two and three orders of magnitude higher than Pu[29]. But Pu-isotope ratios can act as a fingerprint for different sources and information on these ratios can be useful both for identifying sources and for following the migration of Mayak-derived Pu and contaminated soils, sediments, or water bodies.

**TABLE 3**
**Mean Contamination Levels (kBq/m$^2$) in Surface Soils from the Mayak Area[29]**

| Location | Core Depth (cm) | ΣPu (Alpha-Emitting Isotopes) |
|---|---|---|
| **Burial sites** | | |
| Northwest of the repository site | 0–22 | 120 |
| Southeast of the repository site | 0–25 | 71 |
| **Health protection zone (between Lake Karachay and River Mishelyak)** | | |
| 3.6 km southeast of Lake Karachay | 0–21 | 2.4 |
| 4 km south of Lake Karachay | 0–16 | 2.3 |
| **Techa River and Asanov Swamp** | | |
| Asanov Swamp 7 km from Dam 11 | 0–25 | 1.1 |
| | 0–40 | 85 |
| | 0–18 | 10 |
| | 0–14 | 4.7 |
| | 0–20 | 3.3 |
| Techa 15 km from Dam 11 | 0–24 | 5.2 |
| | 0–25 | 7 |
| | 0–20 | 0.4 |
| Muslyomovo 47 km from Dam 11 | 0–10 | 13 |
| | 0–10 | 29 |
| **East Ural Trace** | | |
| 0.5 km southwest of Lake Berdenish | 0–20 | 17 |
| 5 km northwest from Lake Berdenish | 0–30 | 12 |

**TABLE 4**
**Mean Sediment Contamination Densities (Bq/m$^2$) in the Mayak Area[29]**

| Location | Core Depth (cm) | Contamination Density (kBq/m$^2$) |
|---|---|---|
| **Mishelyak River** | | |
| Near borehole | 0–38 | 1.4 |
| **Reservoir 10** | | |
| Northern part of the Reservoir | 0–16 | 470 |
| Southern part of the Reservoir | 0–8 | 510 |
| | 0–20 | 720 |
| **Reservoir 11** | | |
| Northern part of the Reservoir | 0–28 | 42 |
| | 0–40 | 63 |
| Southern part of the Reservoir | 0–19 | 0.2 |
| **Techa River** | | |
| Asanov Swamp (7 km from Dam 11) | 0–42 | 74 |
| Techa (15 km from Dam 11) | 0–29 | 0.5 |
| Techa (25 km from Dam 11) | 0–16 | 0.2 |
| Techa (42 km from Dam 11) | 0–22 | 1.5 |
| Muslyumovo (47 km from Dam 11) | 0–16 | 0.55 |

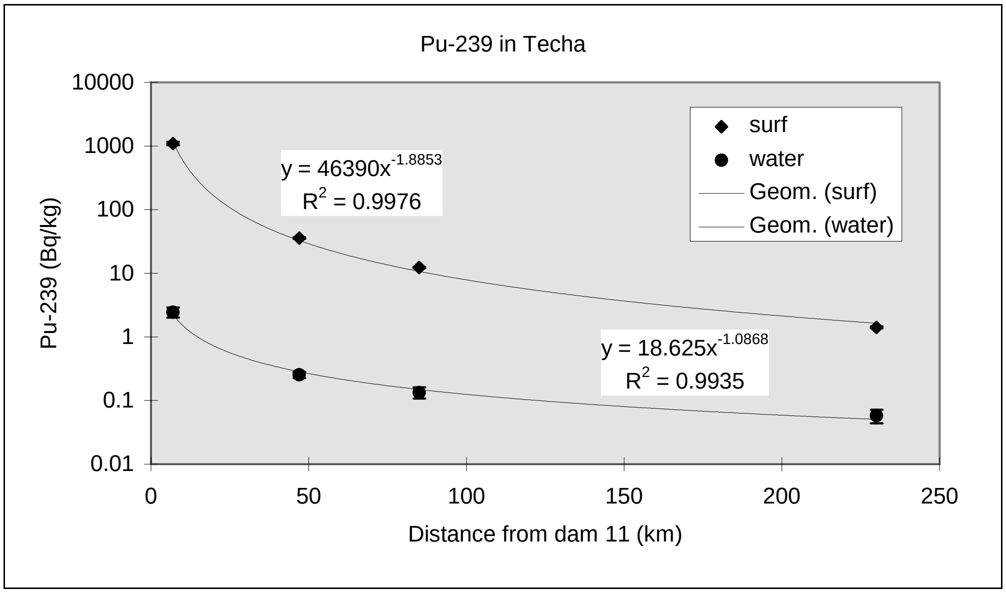

**FIGURE 3**. $^{239}$Pu activity concentrations in surface sediments (Bq/kg) and filtered waters (Bq/l) in the Techa, Iset, and Ob Rivers[30].

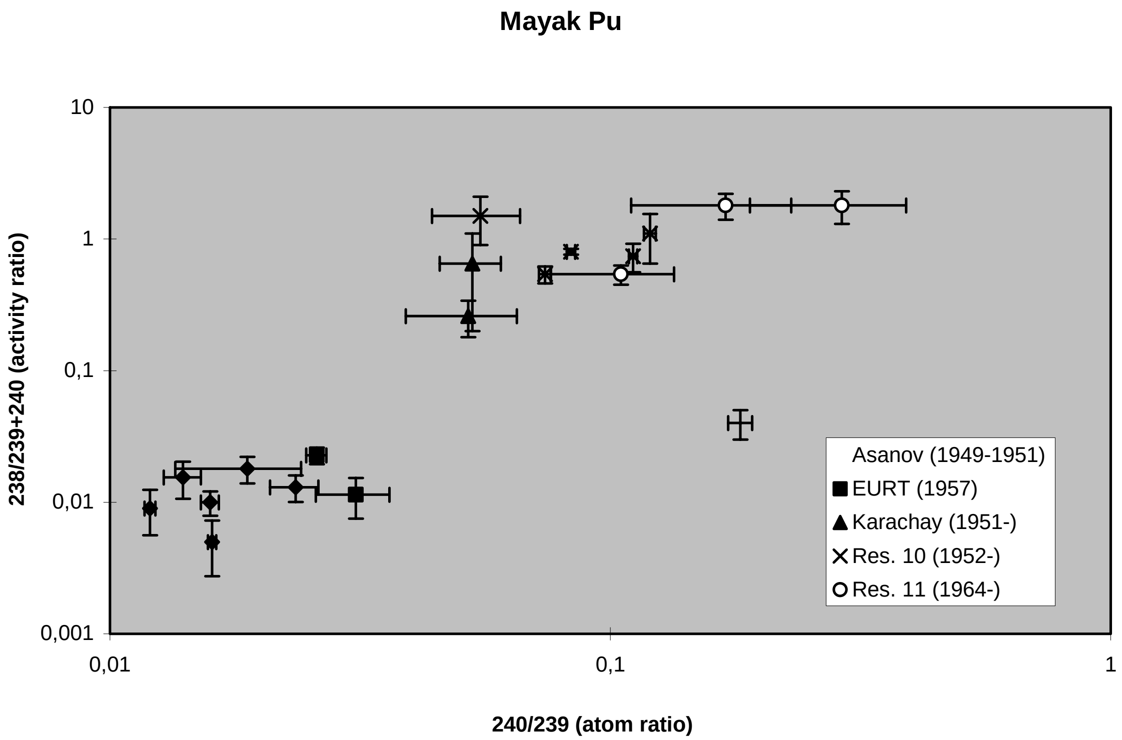

**FIGURE 4.** Two-dimensional plot of $^{240}$Pu/$^{239}$Pu atom ratios and $^{238}$Pu/$^{239+240}$Pu activity ratios in samples from Asanov (n = 6), EURT (n = 2), Karachay Groundwater (n = 2), and Reservoirs 10 and 11 (n = 8). GF = Global Fallout ratio are shown for comparison[30].

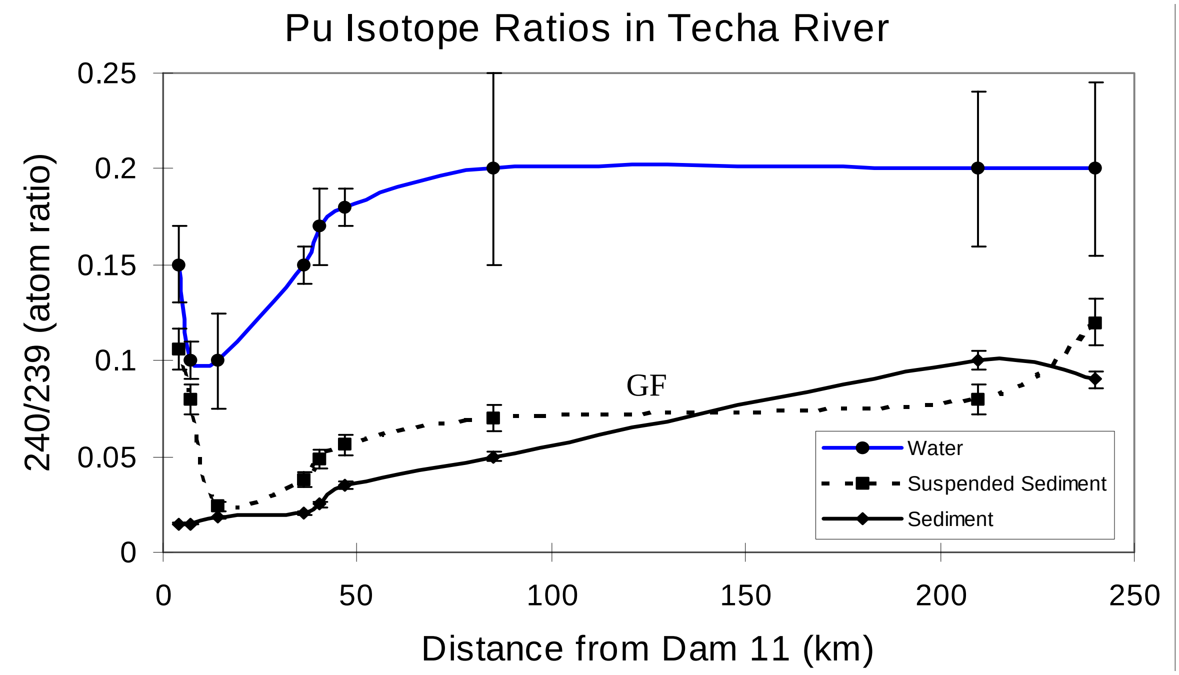

**FIGURE 5**. $^{240}Pu/^{239}Pu$ atom ratios in surface sediments (0–2 cm), suspended sediments (>0.45 μm), and filtered waters (<0.45 μm) in the Techa, Iset, and Ob Rivers[30].

Determination of Pu-isotope ratios in various Mayak samples has identified the presence of different sources and confirmed recent reports of civil reprocessing at Mayak (Fig. 4). Even with only a few samples, clear differences could be seen between the various sites. Activity levels and isotope ratios in soil and sediment samples support the hypothesis that most of the Pu, like other radionuclides in the Techa River, originated from the very early waste discharges to the Techa River between 1949 and 1951. However, enhanced 240/239 ratios in river sediment upper layers (0–2 cm) between 50 and 250 km downstream from the plant indicate a contribution from other, nonfallout sources (Fig. 5)[30].

### Distribution Coefficients Due to Contact Time

The behaviour of Pu in the soil-water system depends on the speciation (Fig. 6). Furthermore, the interaction of Pu-species with soils is a time- and temperature-dependant process. Thus, the distribution coefficient, Bq/kg soil per Bq/l water, increases with time until pseudoequilibrium/ equilibrium is reached ($K_D$).

In a study of $K_D$s for different Pu-species in a Mayak soil-freshwater system, the results show that the association of Pu(III,IV) with soil components occurs rapidly and the retention in soils is about one order of magnitude higher than for Pu(V,VI) and Pu(III,IV)-organic (Fig. 7). When different Pu-species are added to a soil-water system, the interactions follow a two-step reaction. Initial association was faster for Pu(III,IV) than for Pu(III,IV)-organic and Pu(V,VI). Thus, soils act more as a sink for Pu(III,IV) (high $K_D$) than for Pu(III,IV)-organic and Pu(V,VI). Pu(III,IV)-organic and Pu(V,VI) should be considered mobile (low $K_D$).

Time is needed for equilibrium to be established. Until equilibrium is reached, the distribution coefficient can be significantly lower than the established $K_D$ values, which are based on measurement of fallout Pu in sediments and water.

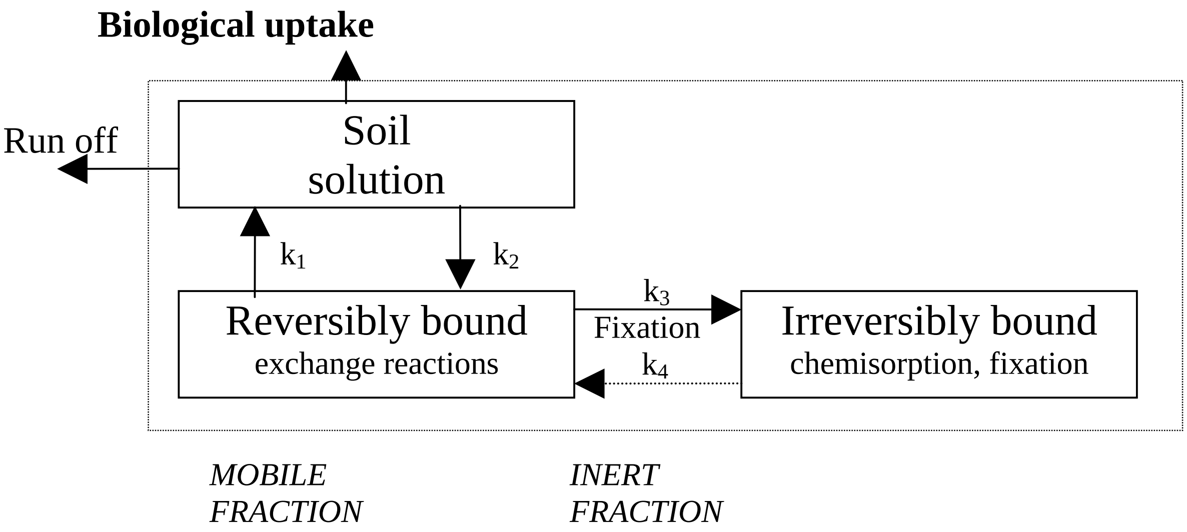

**FIGURE 6**. Processes influencing the radionuclide species in soils[14].

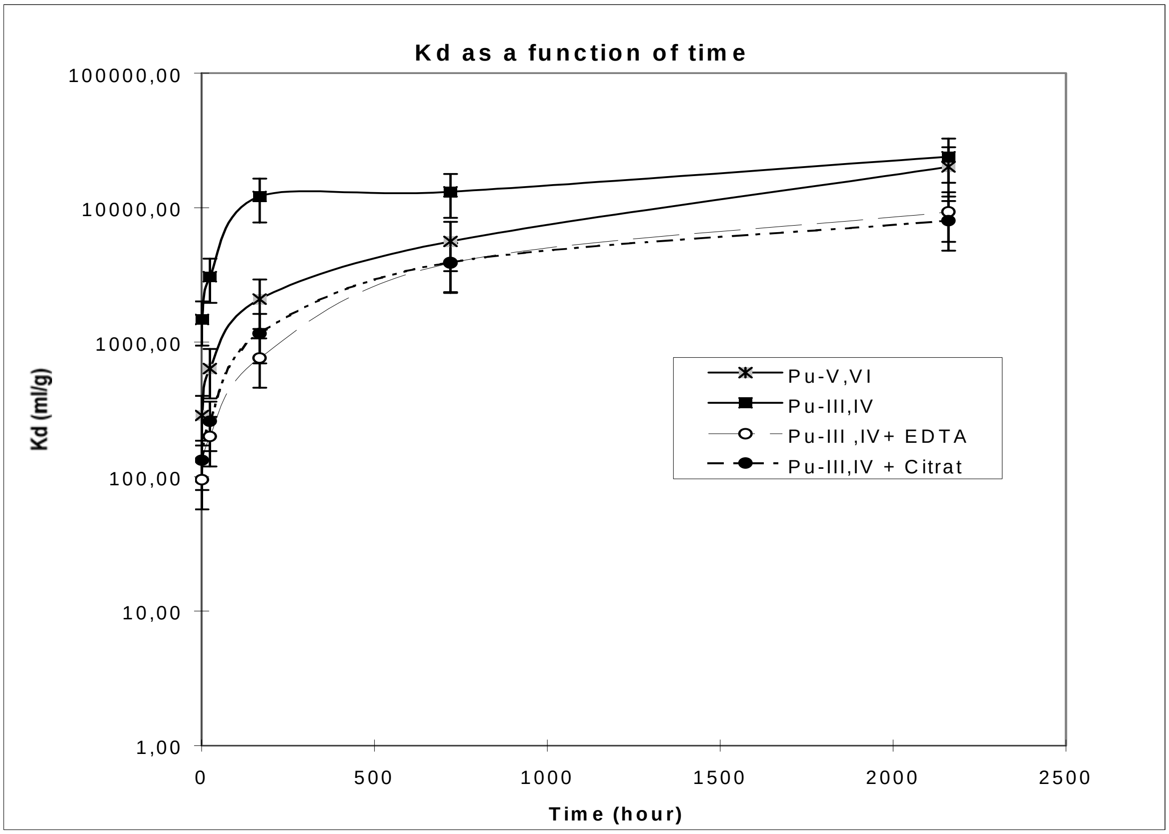

**FIGURE 7**. $K_D$ as a function of contact time: $Pu^{III,IV}$-ionic/colloidal, $Pu^{III,IV}$-organic and $Pu^{V,VI}$[14].

### TRANSPORT OF PU FROM THE IRISH SEA TO THE ARCTIC

Discharges of major transuranium nuclides have fluctuated with peak values of $^{241}Pu$ from 1970–1980[17]. The graph below (Fig. 8) indicates the rate of liquid discharges from 1952–1992.

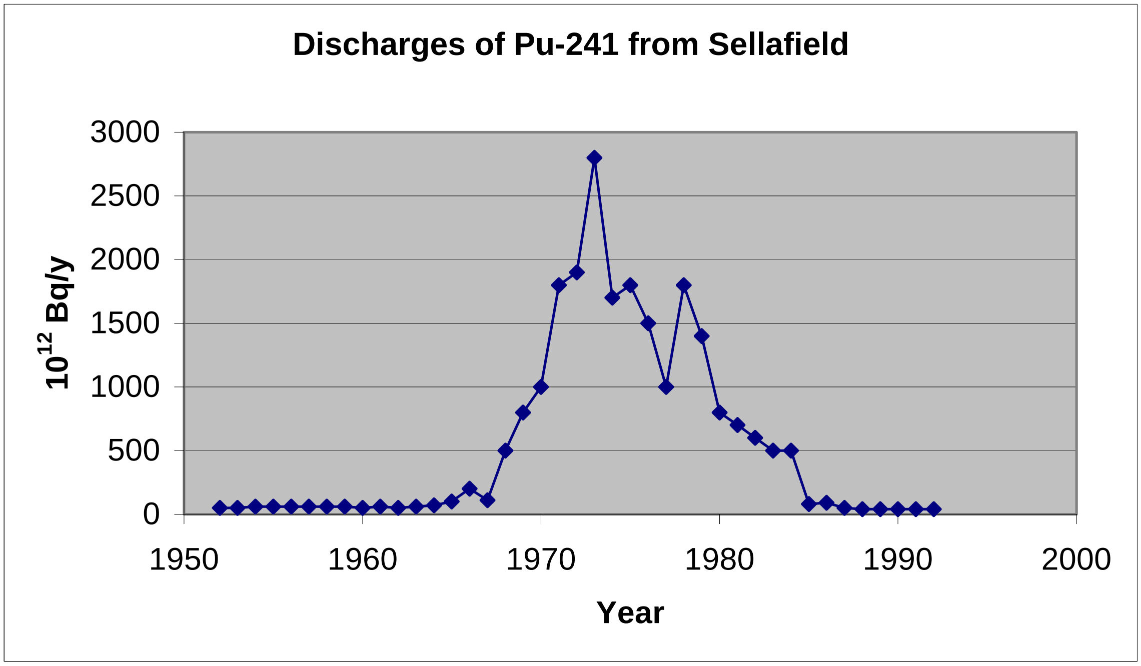

**FIGURE 8**. Discharges of beta-emitting Pu from Sellafield nuclear reprocessing plant, $10^{12}$ Bq/year.

The Pu-alpha discharges were greatest in 1973. Data on $^{238}Pu$, separate from $^{239,240}Pu$, have been available from 1978, since which time the ratio of $^{239,240}Pu/^{238}Pu$ has varied from about 5.5–2.5 to a month-to-month basis[31]. Analysis of surface sediments in the Esk Estuary indicated that this ratio fell from about 20 in 1966 to about 5 or less after 1972[32]. The transit time from Sellafield to the Barents and Greenland Seas is 4–7 years. The yearly average releases from the reprocessing plant during 1973–1976 were 0.04 PBq, 0.011 PBq, and 1.16 PBq for $^{239,240}Pu$, $^{238}Pu$, and $^{241}Pu$, respectively. Transfer factors obtained vary from 0.015–0.033 Bq/m$^3$ (PBq/year)$^{-1}$ for $^{239,240}Pu$, from 0.09–0.036 Bq/m$^3$ (PBq/year)$^{-1}$ for $^{238}Pu$, and from 0.018–0.043 Bq/m$^3$ (PBq/year)$^{-1}$ for $^{241}Pu$ estimated for both particulate and dissolved Pu released. The released dissolved Pu (4%) is readily transported over long distances by currents, with transfer factors between 0.4 and 0.9 Bq/m$^3$ (PBq/year)$^{-1}$[5].

There are no existing discharge data for $^{238}Pu$ for the whole period 1959–1977. By comparing the variety of radionuclide concentrations and isotope ratios in the sediment cores with the available information on decay-corrected discharges, it was possible to estimate the quantity of $^{238}Pu$ discharged in this period to be 94 ± 8 TBq[33]. Discharges of Pu are reduced further from 1993.

About 80% of the Pu from the Sellafield reprocessing plant is removed by sedimentation processes before reaching the Barents and Greenland Seas, a distance of about 4000 km. Resuspension of sediments might contribute to the Pu transport in the future [17].

### Inventory

Concentrations of Pu in surface sediments have not decreased at the same rate as the reductions in the discharge. This is illustrated by the time-series of surface $^{239,240}$Pu concentrations in intertidal mud and saltmarsh from Maryport, Kippford, and Garlieston (Fig. 9), at increasing distance from Sellafield from 1977–1996[34]. Concentrations decreased by a factor of 5 at Maryport during this period, compared with an approximate 2 orders of magnitude decrease in the discharge[6]. At Kippford, there appears to have been a slight decrease since the mid-1980s. Concentrations at Garlieston were relatively constant throughout.

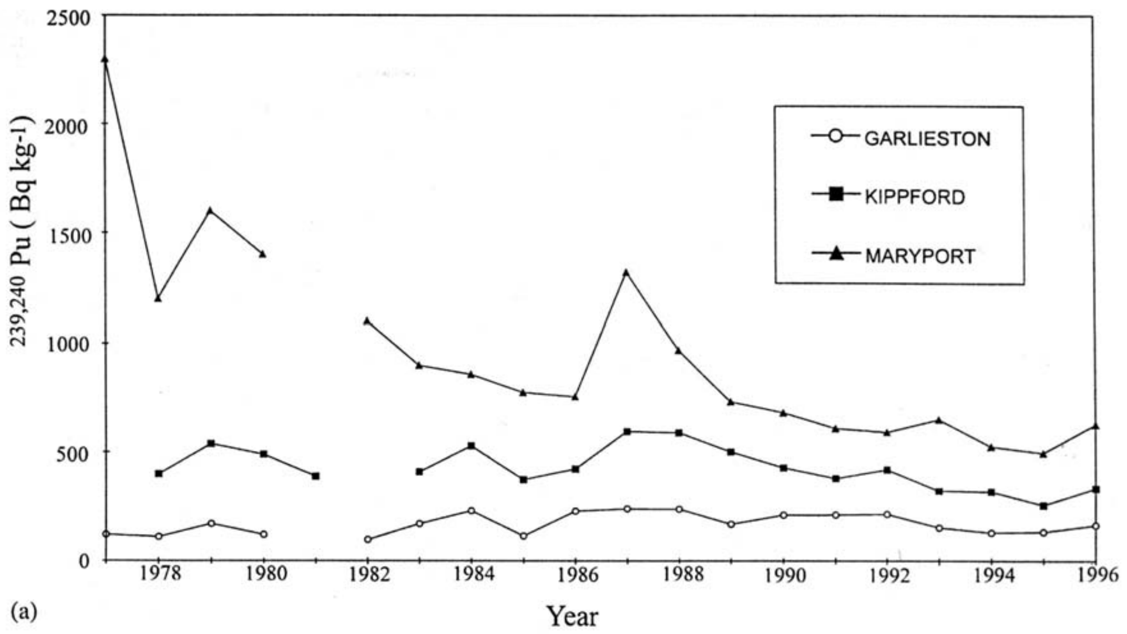

**FIGURE 9.** $^{239,240}$Pu concentrations (Bq/kg dry) in surface intertidal sediments at Maryport, Kippford, and Garlieston, at increasing distance from Sellafield, 1977–1996[34].

The inventories of $^{239,240}$Pu in the subtidal sediments are given in Table 5 together with what this represents as a percentage of the total environmental inventory on the basis of the published discharge data[34].

**TABLE 5**
**Estimated Inventories of $^{239,240}$Pu in Designated Areas in the Subtidal Sediments of the Irish Sea, Expressed as Total Activities (TBq) and as Percentage of the Total, Inputs to the Environment – Based on Hand-Contoured Data from Field Measurement[34]**

| $^{239,240}$Pu Inventory (TBq) | 1978 | % | 1983 | % | 1988 | % | 1995 | % |
|---|---|---|---|---|---|---|---|---|
| All Irish Sea | 248 | 52.3 | — | — | 341 | 58.1 | 360 | 60.8 |
| 1983 Box area | 196 | 41.3 | 236 | 41.2 | 286 | 48.7 | 284 | 47.9 |
| Mud patch area | 115 | 24.2 | 146 | 25.5 | 172 | 29.3 | 151 | 25.5 |

The subtidal sediments of the Irish Sea remain the single most important repository of Pu discharged from the Sellafield site. The proportion of the decay-corrected discharge observed in this compartment has remained relatively constant. Kershaw et al.[17] have seen that the subtidal radionuclide distribution has changed, with a progressive decrease in both surface concentrations and inventories near the Cumbrian coast and increases at more distance sites. This reflects the importance of sediment reworking and transport in influencing the distribution of Pu.

### Oxidation States

In the sea tanks, 99% of the Pu ($\alpha$) was associated with particulate material. The percentage of Pu ($\alpha$) in the pond water effluents retained by filtration was somewhat lower. There were also differences in chemical speciation, with reduced (III + IV) Pu ($\alpha$) predominating in the sea tank effluent and oxidised (V + VI) Pu ($\alpha$) predominating in the filtrate of the effluent from the cooling ponds. In view of the small contribution (<10%) of these pond effluents to the total Pu ($\alpha$) discharges, however, only about 1% of the total would appear to be in an oxidised form[17].

Nelson and Lovett[18] said that due to large differences in $K_D$ values, the initial distribution of Pu between soluble and particulate phases (seabed included) in the sea should be highly dependant on the ratio of oxidation states in the source material. It has been observed that around 5% of the Pu is removed from the area by water transport, probably Pu (VI), and this shows that knowledge of the changes in oxidation state undergone by Pu is important in understanding the ultimate fate of this element in the environment.

## PU IN KARA AND BARENTS SEA

As mentioned above, there have been different sources of contamination of the Kara and the Barents Sea. From 1992–1994, a joint Norwegian-Russian expert group used sonar and remote vehicle in an attempt to find and examine the waste and its surroundings in the Kara Sea. The results show that there is no significant contamination of the Kara Sea, however, high levels of radioactivity in the immediate vicinity of the waste show that there is local contamination at the dump sites. The major risks are for the long term, after the containment corrodes[8,35,36].

There have also been studies done in the area between Franz-Josef Land and Severnaya Zemlya by an Irish team.

### Inventory

New calculated inventories of the dumped reactors have been made[8]. The total activity at the time of dumping is presently estimated by IASAP to be about 37 PBq. The corresponding present time activity (1993/94) has been estimated to be about 4.5 PBq (IASAP-1,5 and 6). Activity of actinides, where Pu will be a major concern, in the dumped reactor components with (6 reactors and the reactors from the ice breaker *Lenin*) and without (6 reactors) spent nuclear fuel is estimated to about 100 TBq.

### Isotopic Ratios

Studies on Arctic marine samples have shown that deviations from global fallout $^{240}Pu/^{239}Pu$ ratios can be identified in sediments collected from dumping sites at Novaya Zemlya. It appears that an influence from low burn-up or noncivil sources is apparent at the Kara Gate, Yenisey Estuary, and Abrosimov Fjord, while the ratios seen in contaminated sediments in Stepovogo Fjord are concordant with high burn-up, civil sources (Fig. 10). Future studies are planned at both the Mayak and Novaya Zemlya sites to obtain more information on the distribution of Pu-ratios at specific sites and provide data on other Pu isotopes and actinides[37].

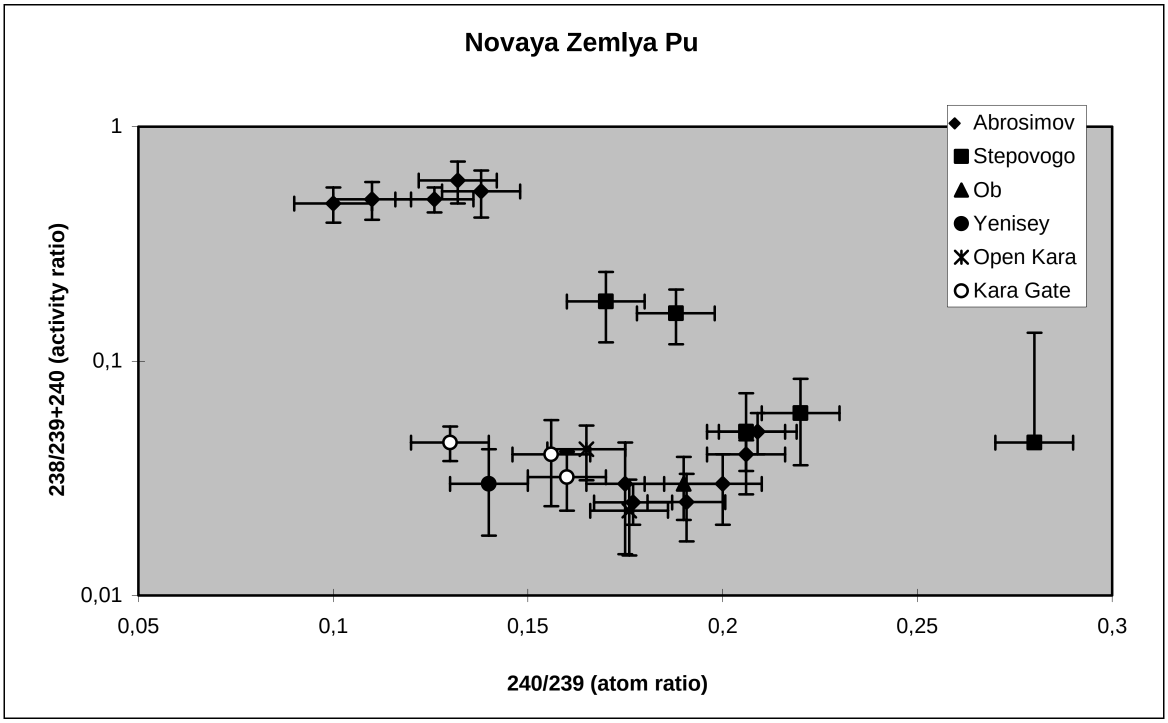

**FIGURE 10**. Two-dimensional plot of $^{240}Pu/^{239}Pu$ atom ratios and $^{238}Pu/^{239+240}Pu$ activity ratios in samples from the Kara Sea and Novaya Zemlya.

### Pu in Subsurface Waters

Mitchell et al.[21] have done some studies of Pu in the Arctic surface and subsurface waters at the St. Anna and Voronin Troughs. The measured Pu concentrations in filtered seawater sampled, taking a transect across the St. Anna and Voronin Troughs extending from 065°E to almost 090°E, are given in Fig. 11. The data confirm that the Pu concentrations in the dissolved phase in this region of the Arctic are extremely low, being in the range of 2.0–8.2 mBq/m$^3$. Concentrations in the surface mixed layer were lowest of all, being <4 mBq/m$^3$ in most cases.

These measurements complement the extensive set of measurements on Pu made in the Norwegian, Barents, and Greenland Seas by Herrmann et al.[38] and a similar set extending across the Arctic Ocean in 1994 by Ellis et al.[39]. These studies revealed a distinct subsurface maximum in the Pu concentration at several hundred metres depth over much of the Arctic Ocean and surrounding areas.

### Distribution Coefficients Due to Contact Time

When radionuclides are released from a source in the marine ecosystem (e.g., radioactive waste in the Stepovogo Fjord)[8,35,36], the assessment of long-term consequences to man depends on information on the source term, transport in water and interactions with sediments ($K_D$), and uptake in marine food products (CF) (Fig. 12).

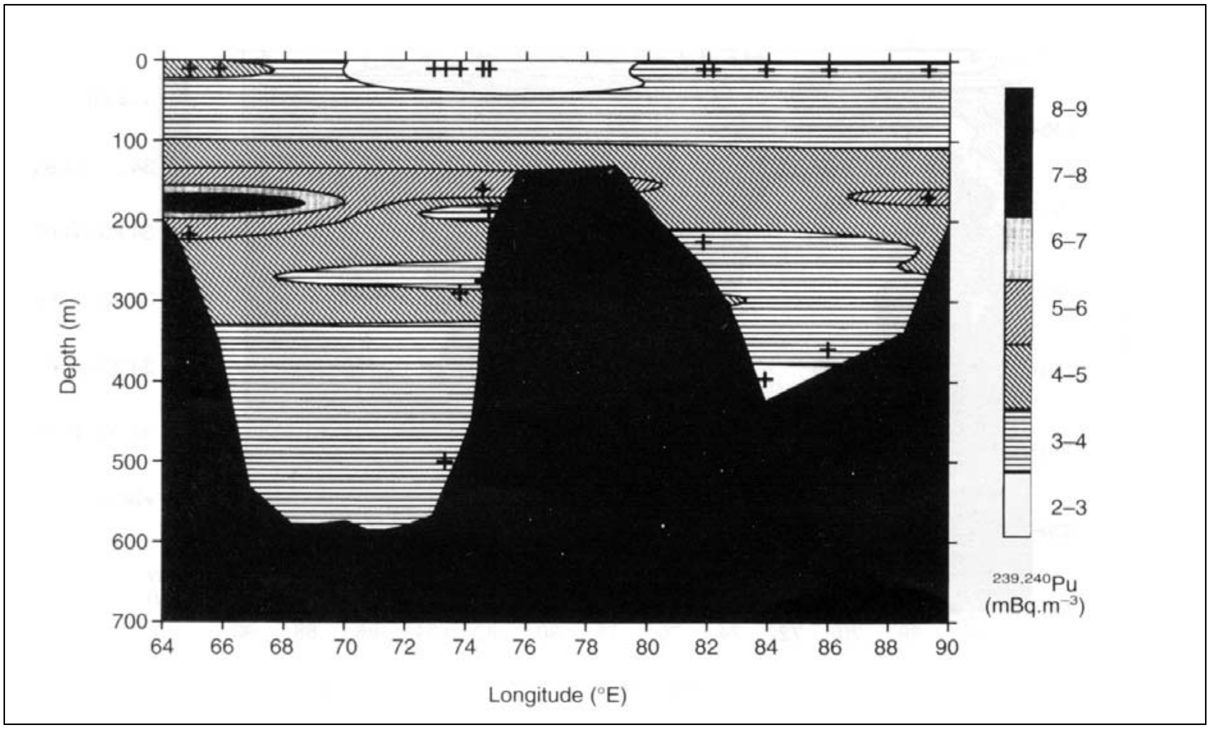

**FIGURE 11**. $^{239/240}Pu$ concentrations in filtered seawater across the St. Anna/Voronin Trough[21].

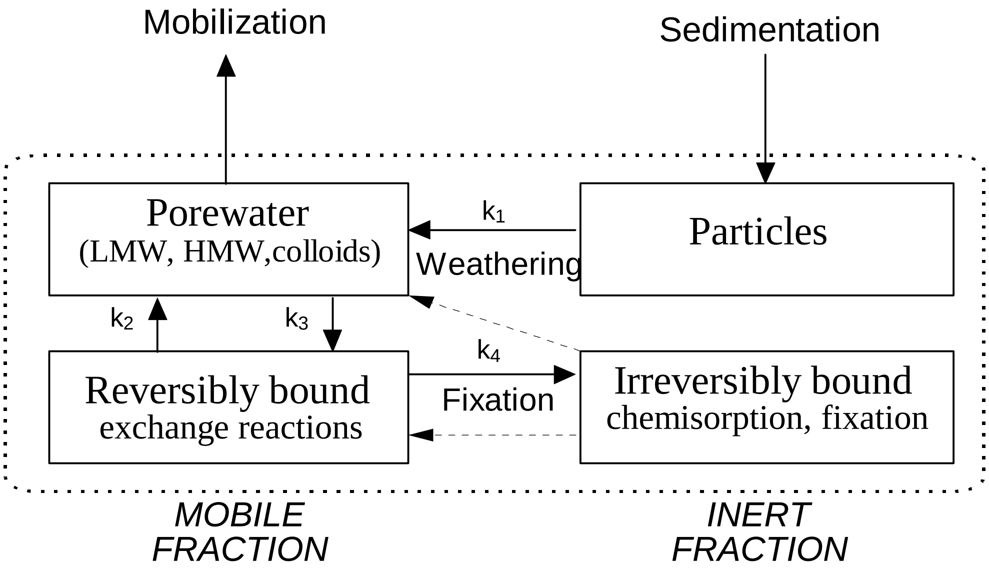

**FIGURE 12**. Processes influencing the radionuclide species in sediments.

Sensitivity analysis of assessment models demonstrates that $K_D$ is one of the most sensitive parameters contributing to large uncertainties in the assessment[40,41]. Information on the source term is usually restricted to the list of inventory and the activity levels. However, the physicochemical forms of released radionuclides are essential for the interaction with sediments and the degree of which sediments will act as a sink or contaminated sediments can act as a diffuse source.

The behaviour of Pu in the sediment-seawater system depends on the speciation. Corrosion particles and aggregating colloids may settle, whilst the retention of mobile Pu-species in sediments may be negligible. Furthermore, the interaction of Pu-species with sediments is a time- and temperature-dependant process. Thus, the distribution coefficient, Bq/kg sediment per Bq/l seawater, increases with time until pseudoequilibrium/equilibrium is reached ($K_D$).

When different Pu-species are added to a sediment-seawater system (sediment from the Stepovogo Fjord, Novaya Zemlja), the interactions follow a two-step reaction, like in the soil-water system mentioned above. The association of Pu(III,IV) with sediment components occurs rapidly and the retention in sediments is about one order of magnitude higher than for Pu(V,VI) and Pu(III,IV)-organic (Fig. 13). Thus, sediments act more as a sink for Pu(III,IV) (high $K_D$) than for Pu(III,IV)-organic and Pu(V,VI). Pu(III,IV)-organic and Pu(V,VI) should be considered mobile (low $K_D$)[15].

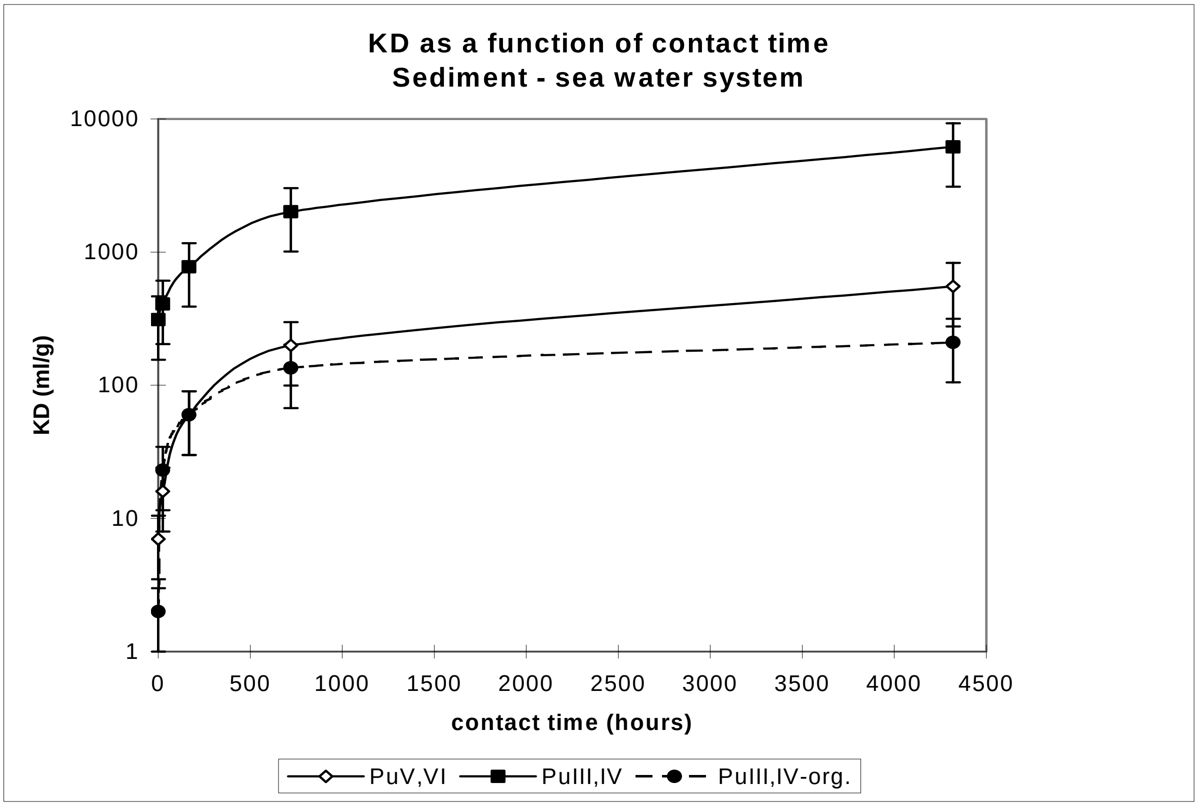

**FIGURE 13**. $K_D$ as a function of contact time: $Pu^{III,IV}$-ionic/colloidal, $Pu^{III,IV}$-organic and $Pu^{V,VI}$[15].

When the temperature is low, time is needed for equilibrium to be established. Until equilibrium is reached, the distribution coefficient can be significantly lower than the mostly used $K_D$ ($10^5$ l/kg), which is based on measurement of fallout Pu in sediments and seawater. Pu speciation and changes in $K_D$ have an important impact on the assessment of collective dose received from a potential release of Pu from fallout, reactor accidents, etc., which shows the need for more detailed studies on speciation and $K_D$s in different sediment-seawater systems. Thus, the contact time between contaminated seawater and sediments should be taken into account in dispersion and dose assessment models.

### Dose Assessment

By implementing the obtained $K_D$s in the NRPA model[42], a preliminary assessment of the collective dose delivered from 1 GBq Pu released to the Ob Bay using different $K_D$-values is about $1.6 \times 10^{-3}$ manSv for Pu(III,IV)-organic and Pu(V,VI), while estimated dose from Pu(III,IV) is about $1.1 \times 10^{-3}$ manSv. All determined from $K_D$s after 6 months of contact time[15].

The NRPA model is an improved version of the Risø-NRPA compartment model with three-dimensional box structure covering the actual ocean space[42]. The model is based on updated information on water mixing in the European coastal waters and the hydrodynamic data used in the model are derived from a three-dimensional World Ocean circulation model.

The model describing a box structure with uniform mixing in all boxes has been improved by implementing dispersion of radionuclides with time, yielding a more realistic approach compared to traditional box modelling and has elements of a more local/regional model[39]. When the mostly used $K_D$ ($10^5$ l/kg) (from fallout Pu) is implemented in the model, the estimated collective dose is about $8 \times 10^{-4}$ manSv, i.e., a factor of about 2 lower than for Pu(III,IV)-organic and Pu(V,VI) (Fig. 14)[15].

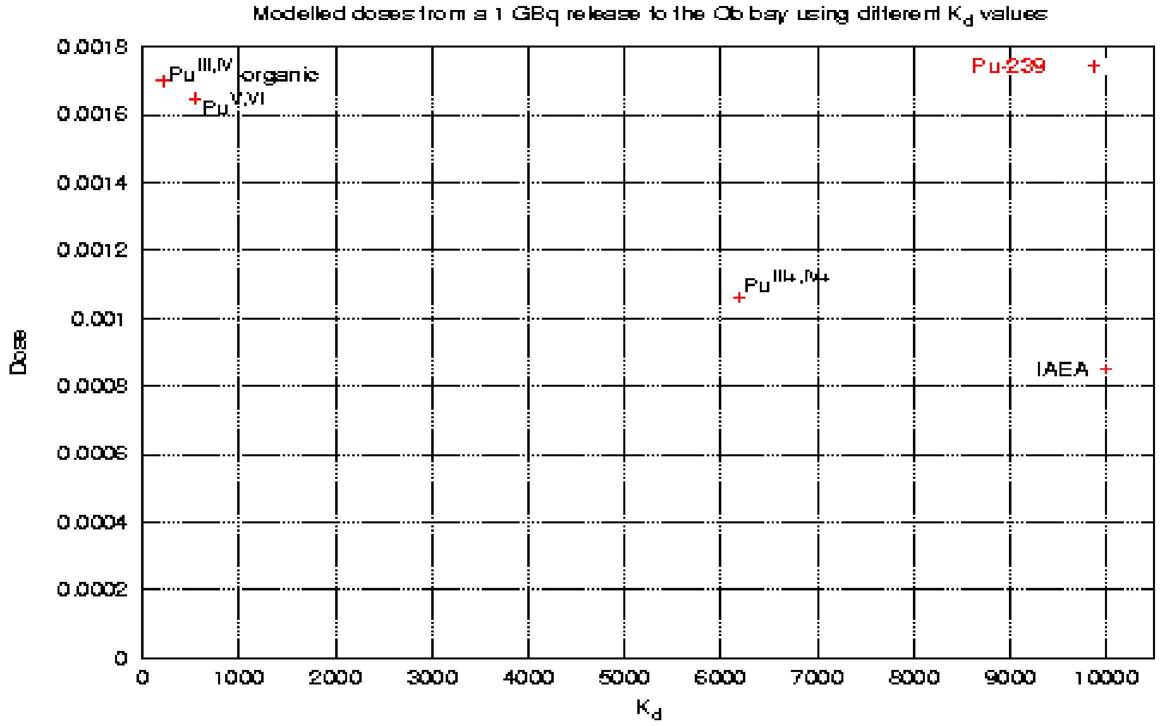

**FIGURE 14**. Modelled doses from a 1 GBq released to the Ob Bay using the different $K_D$s for the different Pu species after a contact time of 6 months. $K_D$s are given in l/kg and doses are given in manSv[15].

### PU IN OB AND YENISEY ESTUARIES AND YENISEY RIVER

The Ob and Yenisey influence is particularly interesting as both rivers have weapons-grade Pu sources in their catchment areas, including the Russian Pu production and reprocessing plants at Mayak, Tomsk-7, and Krasnoyarsk and the Semipalantinsk nuclear weapons testing site in Kazakhstan, and together represent the largest input of freshwater to the Arctic area. Hence, measurement of the Pu-isotope ratios offers both a means of identifying the origin of radionuclide contamination and the influence of the various nuclear installations on inputs to the Arctic, as well as a potential method for following the movement of water and sediment loads in the rivers. The river systems represent a source of a variety of pollutants to the Arctic and information on their environmental status and behaviour is of interest to various scientific disciplines[43].

The data indicated a clear influence from a low $^{240}Pu/^{239}Pu$ source in surface sediments collected from the Yenisey Estuary (Table 6). Previous studies have indicated similar results, although based on much fewer samples than the present study[38,44]. The results also show an increase in Pu concentration and a decrease in isotope ratio going upstream from the estuary; the sample collected from the site closest to the Krashnoyarsk site having the highest concentration (11.02 ± 0.09 mBq/g $^{239,240}Pu$) and lowest observed $^{240}Pu/^{239}Pu$ isotope ratio (0.049 ± 0.001)[44]. Measurements of Pu isotope ratios in Kara Sea and Ob sediments gave no evidence for significant nonglobal fallout sources, which is in agreement with published data, again on a much lower number of samples. Finally, it should be stressed that the concentration level of Pu in both estuaries is low and many orders of magnitude below that reported in the river catchment sites close to the nuclear installations (Table 6)[45].

**TABLE 6**
**Summary of Results of Measurements in River Yenisey and the Ob and Yenisey Estuaries, Together with Possible Sources of Contamination[29,30,38,45,46,47]**

| | n | $^{239,240}Pu$ Bq/kg Average | $^{239,240}Pu$ Bq/kg Range | $^{240}Pu/^{239}Pu$ Atom Ratio Average | $^{240}Pu/^{239}Pu$ Atom Ratio Range | Sources | Total Pu Released | Pu at Site (Bq/m$^2$) |
|---|---|---|---|---|---|---|---|---|
| Ob Estuary | 7 | 0.40 ± 0.23 | 0.16 – 0.75 | 0.17 ± 0.02 | 0.16 – 0.18 | Mayak | | |
| | | | | | | River Techa | 0.002 PBq | $1 \times 10^3 – 7 \times 10^5$ |
| | | | | | | Lake Karachay | 1.5 PBq | |
| | | | | | | Tomsk-7 | | |
| | | | | | | Basins, Reservoir | $4.8 \times 10^3$ PBq | |
| | | | | | | Geological formations | $3.7 \times 10^4$ PBq | |
| | | | | | | Semipalatinsk | | $1 \times 10^2 – 1 \times 10^3$ |
| | | | | | | Ground Zero | | $1 \times 10^4 – 3 \times 10^5$ |
| | | | | | | Balapan | | |
| Yenisey Estuary | 8 | 0.24 ± 0.19 | 0.003 – 0.61 | 0.11 ± 0.01 | 0.09 – 0.13 | Krasyonarsk | $5 \times 10^3$ MBq[a] | |
| Yenisey River | 5 | 3.7 ± 4.2 | 1.0 – 11.0 | 0.08 ± 0.03 | 0.05 – 0.12 | | | |
| Kara Sea | 7 | 0.55 ± 0.27 | 0.14 – 0.96 | 0.17 ± 0.01 | 0.15 – 0.18 | Novaya Zemlya | 5 – 10 TBq | |
| Global Fallout | | | | | 0.17 – 0.19 | | 100 – 400 TBq | |

[a]Data from 1996–2000

### CONCLUSION

Anthropogenic plutonium has been introduced into the environment over the past 50 years as the result of the detonation of nuclear weapons and operational releases from the nuclear industry. In the Arctic environment, the main source of plutonium is from atmospheric weapons testing, which have resulted in a relatively uniform, underlying global distribution of plutonium, but previous studies of plutonium has shown that, at certain sites, other releases have given rise to enhanced local concentrations. Studies on Arctic marine samples have shown that deviation from global fallout $^{240}Pu/^{239}Pu$ ratios can be identified in sediments collected from dumping sites at Novaya Zemlya. It appears that an influence from low burn up or non-civil sources is apparent at the Kara Gate, Yenisey Estuary and Abrosimov Fjord, while the ratios seen in contaminated sediments in Stepovogo Fjord are concordant with high burn-up, civil sources. Also a study in Ob and Yenisey Estuaries has shown conclusively that deviation from global fallout $^{240}Pu/^{239}Pu$ ratios can be identified in sediments from the Yenisey River and Estuary. An influence from low burn-up or non-civil sources is apparent in the Yenisey Estuary, whereas plutonium in the Ob Estuary is dominated by global fallout.

The overall data suggest that the plutonium-isotope "fingerprint" would be a useful method of following the migration and behaviour of both plutonium and sediments and particle loads in the Arctic environment, to obtain more information on the distribution of and mobility of plutonium associated with Sediment samples.
